# Different Methylation of CpG-SNPs in Behcet's Disease

**DOI:** 10.1155/2019/3489305

**Published:** 2019-05-16

**Authors:** Yang Huang, Handan Tan, Qingfeng Cao, Gangxiang Yuan, Guannan Su, Peizeng Yang

**Affiliations:** The First Affiliated Hospital of Chongqing Medical University, Chongqing Key Laboratory of Ophthalmology and Chongqing Eye Institute, Chongqing, China

## Abstract

**Purpose:**

We recently performed an Epigenome-Wide Association Studies (EWAS) study in Behcet's disease (BD) and identified various cytosine–phosphate–guanine (CpG) loci that were aberrantly methylated. In the current study, we wanted to investigate whether these sites contained genetic polymorphisms and whether the frequency of these polymorphisms was altered in BD.

**Methods:**

A two-stage study was performed. The first stage involved 358 BD patients and 704 healthy controls to investigate genetic variants of 10 CpG-SNPs (rs10454134, rs176249, rs3808620, rs10176517, rs11247118, rs78016579, rs9461624, rs10492166, rs34929465, and rs6507921) using an iPLEX Gold genotyping assay and a Sequenom MassARRAY. In the second stage, an additional 172 independent BD patients and 330 healthy individuals are to confirm trends found in the first stage.

**Results:**

A higher frequency of both the rs10454134 AG genotypes (p = 0.008, OR = 1.413, 95% CI = 1.094-1.826) and a lower GG genotype frequency (p = 0.003, OR = 0.630, 95% CI = 0.465-0.854) were found in BD patients compared to the controls in the first stage. However, after correcting for multiple comparisons, all associations identified in the first stage lost statistical significance. The frequencies of the other CpG-SNPs investigated were not different between BD patients and controls. The second stage was designed using an additional cohort to confirm the association with CpG-SNP, rs10454134. The data failed to confirm the association between this CpG-SNP and BD.

**Conclusions:**

This study did not show an association between BD and CpG-SNPs in gene sites that were earlier shown to be aberrantly methylated.

## 1. Introduction

Behcet's disease (BD) is a chronic, relapsing, and multisystemic inflammatory disorder. Its classical clinical characteristics include oral aphthae, genital ulcers, multiform skin lesions, and recurrent uveitis with hypopyon [1]. Its etiology and pathogenesis are not yet fully understood. Generally, it is thought that BD is caused by the interaction of genetic variation and environmental factors. To date, there are numerous genes shown to be associated with BD [2–4]. DNA methylation is a pivotal part of the epigenome and provides highly complementary data on the regulation of genomic regions [5, 6]. Previous study proved that epigenetic modification of some genes dynamics is involved in the pathogenesis of BD, such as cytoskeletal [7]. Furthermore, aberrant changes in DNA methylation have been shown to lead to abnormal gene expression in the pathogenesis of BD, including IL6, IL10, SOCS1, IRF8, GATA3, and TGF-*β* [8–12].

Sequence variations can change the CpG, which may result in indifferences in DNA methylation between individuals. Cytosine–phosphate–guanine single nucleotide polymorphisms (CpG-SNPs) are point mutated CpG sites [13] and may play a role in the methylation status of this gene region. DNA methylation is an important gene silencing mechanism and may play a role in controlling pathways of inflammation [14]. CpG-SNPs in the promoter regions have been found to be associated with various disorders, including type 2 diabetes [15], breast cancer [16], coronary heart disease [17], and psychosis [18]. However, SNPs located in CpG sites in patients with autoimmune diseases such as BD have not been reported and were therefore the subject of the study described here. Functional CpG-SNPs with an aberrant methylation status were selected from the Epigenome-Wide Association Study (EWAS) we performed earlier [19], and genotype frequency was compared between BD patients and healthy controls.

## 2. Materials and Methods

### 2.1. Study Population

The study recruited a total of 530 BD patients and 1034 healthy individuals (Table 1), visiting the uveitis center of the First Affiliated Hospital of Chongqing Medical University from January 2009 to September 2017. All individuals were Chinese Han and all BD patients had uveitis. BD patients and controls were matched according to race which all from Chinese Han and geography. Healthy individuals who had systemic immune diseases or other chronic diseases were excluded. Diagnosis of BD was made according to the standard International Study Group for BD, requiring the presence of oral ulceration as well as any two of the following symptoms: genital ulceration, uveitis, multiform lesions, or a positive pathergy test [20]. The local research ethics committee of the First Affiliated Hospital of Chongqing Medical University (permit no. 2009-201008) approved the study. A written informed consent was obtained from all participants which abided by the tenets of the Declaration of Helsinki.

### 2.2. CpG-SNPs Screening

Target CpG-SNPs were selected according to our previous EWAS results [19] that included 60 BD patients and 60 matched healthy controls using the Illumina Human Methylation450K platform (Illumina, San Diego, CA, USA). A series of screening criteria was used to find target sites including a CpG-SNP methylation p value less than 0.05 and a Beta.Difference either less than -0.14 or more than 0.14 or p value<10^−5^. Beta values used to score the methylation level ranging from 0 (unmethylated) to totally methylated [21, 22]. In addition, the minor allele frequency (MAF) in the Chinese Han population needed to be greater than 0.05, excluding the sites not included in the Han Chinese Hap Map database (https://www.ncbi.nlm.nih.gov/snp/). Furthermore, according to the UCSC (GRCh37/hg19) and HaploReg v4.1 (http://pubs.broadinstitute.org/mammals/haploreg/haploreg.php) databases [23, 24], the CpG-SNP sites located in potential functional regions (promoter region, enhancer region, CCCTC-binding factor (CTCF) binding region, first exon region, 5′UTR, TSS1500) [25] were selected. Finally, linkage disequilibrium (LD) data from the Han Chinese Hap Map database were also used to exclude SNPs in LD with each other. In total, 10 CpG-SNPs were selected based on these criteria and included rs10454134, rs176249, rs3808620, rs10176517, rs11247118, rs78016579, rs9461624, rs10492166, rs34929465, and rs6507921 (Table 3).

### 2.3. DNA Extraction and Genotyping

The two experimental groups including both BD individuals and healthy controls donated peripheral blood, which was applied to extract genomic DNA extraction with a QIAmp DNA Blood Mini Kit (Qiagen Inc., Valencia, CA, USA) and stored in 3.2% sodium citrate-treated tubes at -80°C. The iPLEX Gold genotyping assay and Sequenom MassARRAY (Sequenom, CA, USA) were performed to identify genotype of the 10 CpG-SNPs. iPLEX reactions primers were designed by SNP Assay Design software (version 3.0) (Table 2). All procedures were performed according to manufacturer's instructions.

### 2.4. Statistical Analysis

The healthy controls should satisfy Hardy–Weinberg equilibrium (HWE) (p value<0.05). Hardy-Weinberg equilibrium analysis was using chi-square (*χ*2) test, while the genotype frequency was evaluated by direct counting. Fisher's exact test or *χ*2 test was applied to estimate the differences in the allele and genotype frequencies of all CpG-SNPs between BD patients and healthy controls. P value was carried out by SPSS (version 17.0; SPSS Inc., Chicago, IL). Multiple comparisons were performed to correct P-value by using the Bonferroni method, whereby the p value was multiplied with the number of comparisons (P corrected (Pc)). When Pc< 0.05, it was considered to be significant.

## 3. Results

### 3.1. Clinical Features of the Subjects

The demographics and clinical symptoms of the BD patients and the healthy controls are displayed in Table 1. The healthy cohort consisted of 506 men and 528 women, who were on average 39.3 ± 10.5 years old. The BD patients comprised of 435 men and 95 women, with an average age of 34.3 ± 9.6.

### 3.2. CpG-SNPs Selected

SNPs located in the CpG loci with a CpG-SNP methylation level with a p value of < 0.05 and a Beta.Difference either less than -0.14 or more than 0.14 and MAF>0.05 were identified and eleven CpG-SNPs were included, according to the following criteria: (1) the MAF in the Chinese Han population was greater than 0.05 (https://www.ncbi.nlm.nih.gov/snp/); (2) CpG-SNP sites are located in potential functional regions according to UCSC (GRCh37/hg19) and HaploReg v4.1 (http://pubs.broadinstitute.org/mammals/haploreg/haploreg.php) databases [23, 24]; (3) linkage disequilibrium (LD) data from the Han Chinese Hap Map database was considered. Three CpG-SNPs were chosen: rs78016579, rs34929465, and rs6507921. The methylation levels of CpG-SNPs were considered with any sites with a p value<10^−5^ and the MAF of SNP in the CpG site should be greater than 0.05. According to these criteria, 87 CpG-SNPs were included. However, after these CpG-SNPs were subjected to the aforementioned criteria, only 7 CpG-SNPs (rs10454134, rs176249, rs3808620, rs10176517, rs11247118, rs9461624, and rs10492166) were included in the study. Therefore, a total of 10 CpG-SNPs were included in this study (Table 3).

### 3.3. Genotyping of the CpG-SNPs in BD

Nine CpG-SNPs (rs10454134, rs176249, rs3808620, rs10176517, rs11247118, rs78016579, rs9461624, rs10492166, and rs6507921) were genotyped successfully in 370 BD patients and 704 controls. One CpG-SNP, rs34929465, was excluded, since it could not be analyzed successfully. Seven SNPs found in the healthy controls met the Hardy-Weinberg equilibrium and two SNPs (rs11247118, rs78016579), that deviated from the Hardy–Weinberg equilibrium (p-value<0.05), were excluded. Uncorrected p values only showed a significant association of rs10454134 with BD (Table 4). A higher frequency of both the rs10454134 AG genotypes (p = 0.008, OR = 1.413, 95% CI = 1.094-1.826) and a lower GG genotype frequency (p = 0.003, OR = 0.630, 95% CI = 0.465-0.854) were found in BD patients compared to the controls. After correcting for multiple comparisons, these associations lost statistical significance. To further confirm the trend observed for the CpG-SNP, rs10454134, 172 additional independent BD patients and 330 healthy individuals were recruited. This second stage study also failed to demonstrate an association between this CpG-SNP and BD, even after combining both cohorts (Table 5). Stratified analyses were performed to investigate whether these CpG-SNPs might show an association with the primary clinical features. We chose genital ulcers since this feature has a frequency of approximately 50% in our BD cohort. After Bonferroni correction, no association was observed after stratification by genital ulcers.

## 4. Discussion

In this study, we failed to find an association between functional CpG-SNPs and BD. Functional CpG-SNPs were selected from data obtained in a previous study, whereby we identified various CpG sites with a different methylation status in BD patients [19]. The study presented here expanded these findings and investigated whether genetic polymorphisms in these sites might affect predisposition to BD. The fact that no association could be detected suggests that methylation of these sites may not be dependent on genetic variation of these sites themselves but may be regulated by other mechanisms. Further studies are needed to elucidate the exact mechanisms involved.

BD is considered an autoimmune or autoinflammatory disorder, characterized by chronic and recurrent episodes of posterior or panuveitis. It is generally thought that BD is caused by the combination of genetic variants and environmental factors. Accumulated evidences in previous studied have unfolded epigenetic control of IL6, IL10, SOCS1, IRF8, GATA3 and TGF-*β* expression participates in the pathogenesis of BD [8–12]. The introduction or removal of CpG dinucleotides (possible sites of DNA methylation associated with the environment [6]) has been suggested as a potential mechanism through which SNPs can influence gene transcription and expression via epigenetics [26–29]. Many studies have reported that CpG-SNPs are associated with different diseases, such as type 2 diabetes, breast cancer, coronary heart disease, and psychosis which show a clear interaction between genetic (SNPs) and epigenetic (DNA methylation) regulation [15–18]. The role of SNPs located in CpG sites in autoimmune disease has not been widely addressed and we therefore expanded our earlier studies in this area. We chose BD, since this is a uveitis entity that is commonly observed in China allowing sufficiently large sample sizes to achieve adequate statistical power and allow meaningful conclusions. Identification of genetic variants may lead to novel therapies even in the absence of direct knowledge of the pathogenetic mechanisms involved.

A previous study, where a CpG-SNP MWAS (methylome-wide association studies) was performed, showed that CpG-SNP rs3796293 reached methylome wide significance in psychosis [18]. Rs3796293 is located in the gene encoding for interleukin 1 receptor accessory protein and supports the role of local inflammation in the pathogenesis of psychosis. In a genome wide association study (GWAS), rs3796293 was not found to be associated with the disease, suggesting that methylation of rs3796293 may not be the directly related to genetic variation of this site [18]. This result is consistent with our study in that genetic variation of significant methylation loci in BD did not show an effect on disease risk.

This study has several limitations. As SNPs were only selected if they satisfied the defined criteria, other unknown functional SNPs in CpG loci with a potential association with BD may have been missed and need to be investigated in future studies. Despite the fact that we had a large cohort of patients, it is possible that a weak but significant association might have been missed. The gene frequency of the SNPs analyzed in our study has only been tested in normal populations and no reference is available in clinical disease. It should also be noted that methylation changes at certain loci may be the result of the disease rather than its cause.

## 5. Conclusion

In conclusion, this study did not show an association between BD and CpG-SNPs in sites that have an aberrant methylation status.

## Figures and Tables

**Table 1:** Clinical features, age, and sex distribution of BD patients and healthy controls.

Clinical Features	Total	%
*Patients with BD* ^*a*^	530	
Mean age ± SD^b^	34.3 ± 9.6	
Male	435	82.1
Female	95	17.9
Uveitis	520	98.1
Oral ulcer	530	100.0
Genital ulcer	286	54.0
Skin lesion	395	74.5
Arthritis	108	20.4
Pathergy reaction	65	12.3
*Controls*	1034	
Mean age ± SD	39.3 ± 10.5	
Male	506	48.9
Female	528	51.1

a: BD = Behcet's disease,

b: SD = standard deviation;

**Table 2:** Primers used in the analysis of restriction fragment length polymorphism (RFLP) in CpG-SNPs.

	1st-PCRP	2nd-PCRP	UEP_SEQ
rs10454134	ACGTTGGATGAACCAGTTACTCAGACTGCG	ACGTTGGATGGTAGAACCTCGTATCACCAC	ATCACTGAATTTGCTCCG
rs176249	ACGTTGGATGTACCTTCAGTGGCAGTCTAC	ACGTTGGATGCAGATTTGAATGGAGCTCCC	GAGCGCCCTCCAGCATGGCTTGCC
rs3808620	ACGTTGGATGCAGTAACGAGAGTAAATGGG	ACGTTGGATGATGGCGCTCTGAGTACTTTC	ACTTTCTGTCTCAGTCC
rs10176517	ACGTTGGATGGAGAACAGAAGCCTAGTTCA	ACGTTGGATGGTAAAGCCTTACTTTTTCCC	CCTTTAAGAGAAAGAGAGGTC
rs11247118	ACGTTGGATGGTACATGTTGTTCTCCCTGC	ACGTTGGATGCCTGAGGATGTGTCTCATAC	TGTGTCTCATACTCAAACT
rs78016579	ACGTTGGATGACCTTTACCTACTGAACCTG	ACGTTGGATGACAGGAACCAGCTAGTTACC	GTGAAACAATGAGTAGAATGTGA
rs9461624	ACGTTGGATGCATTTCTCATGAGTCAGCGG	ACGTTGGATGATAGAGGGGCTGTGTCATAG	TGTGTCATAGACGTCCGAC
rs10492166	ACGTTGGATGGCTATCCTACTTTGGAGAAG	ACGTTGGATGCTTCCTCTTTTAGTTCAGGC	AGGCATATATTCTCTGGC
rs34929465	ACGTTGGATGTGGACAATCCCGAAATTGGC	ACGTTGGATGTACCCATTACCGTCCTTTCC	CATCCTTCTACCACATGC
rs6507921	ACGTTGGATGTCCCAAAGATCCTTAGACGG	ACGTTGGATGCCAAACCTTCTAAGCCATGC	AGAAATTTTATCATCAAGACTTC

**Table 3:** SNPs located in differential methylation CpG sites from EWAS in BD.

Target_ID	P.Value	Beta.Difference	ADDRESSA_ID	CHR^a^	UCSC_REFGENE_NAME^b^	Functional region	CpG-SNP	MAF^c^
cg10181180	3.65E-13	-0.033524043	39741415	2		Promoter	rs10454134	0.230
cg15835500	3.61E-12	-0.035509286	51694401	6		CTCF-binding	rs176249	0.276
cg12480843	1.8E-11	-0.037228341	23659445	8	UBE2W	TSS200;TSS200^d^	rs3808620	0.184
cg08338478	1.3E-10	-0.042262334	10617308	2	HECW2	Enhancer	rs10176517	0.162
cg00062736	3.55E-10	-0.086936418	63727392	15	MEF2A	Enhancer	rs11247118	0.392
cg23296792	0.000000856	-0.175644181	72682370	5	FBXO38	Enhancer	rs78016579	0.135
cg03157605	0.000000902	-0.028707179	53675427	6	MDC1	1stExon;5′UTR^e^	rs9461624	0.059
cg16744961	0.000103889	-0.061269519	60757411	12	CLECL1	TSS200	rs10492166	0.424
cg26824678	0.002223998	-0.155996614	49786417	1	CAPZB	Promoter	rs34929465	0.258
cg00254095	0.004197255	-0.141190315	69780487	18	RPL17;SNORD58C;U58	TSS1500	rs6507921	0.478

a: CHR = Chromosome containing the CpG;

b: UCSC_REFGENE_NAME = Target gene name(s), from the UCSC database;

c: MAF = Minor allele frequency of SNP(s);

d: TSS = transcription start site;

e: UTR = untranslated region

**Table 4:** Genotype and allele frequencies of CpG-SNP polymorphisms in BD and healthy controls (first stage study).

Target_ID	CpG-SNP	Genotype	BD n (%)	Controls n (%)	P value	Pc value	OR	95% CI
cg10181180	rs10454134	total sample	*355*	*704*				
		AA	87 (0.245)	173 (0.246)	0.981	NS	0.996	0.741-1.340
		AG	195 (0.549)	326 (0.463)	0.008	NS	1.413	1.094-1.826
		GG	73 (0.206)	205 (0.291)	0.003	NS	0.630	0.465-0.854
		A	369 (0.520)	672 (0.477)	0.065	NS	1.185	0.989-1.420
		G	341 (0.480)	736 (0.523)			0.844	0.704-1.011
cg15835500	rs176249	total sample	*357*	*702*				
		AA	13 (0.036)	25 (0.036)	0.947	NS	1.023	0.517-2.025
		AG	119 (0.333)	207 (0.295)	0.200	NS	1.196	0.910-1.572
		GG	225 (0.630)	470 (0.670)	0.203	NS	0.841	0.645-1.098
		A	145 (0.203)	257 (0.183)	0.266	NS	1.137	0.906-1.427
		G	569 (0.797)	1147 (0.817)			0.879	0.701-1.103
cg12480843	rs3808620	total sample	*355*	*704*				
		GG	339 (0.955)	670 (0.952)	0.815	NS	1.075	0.585-1.976
		CG	16 (0.045)	33 (0.047)	0.895	NS	0.960	0.521-1.768
		CC	0 (0.000)	1 (0.001)	0.477	NS	/	/
		G	694 (0.977)	1373 (0.975)	0.742	NS	1.106	0.608-2.012
		C	16 (0.023)	35 (0.025)			0.904	0.497-1.645
cg08338478	rs10176517	total sample	*354*	*703*				
		CC	190 (0.537)	415 (0.590)	0.096	NS	1.244	0.962-1.609
		CT	145 (0.410)	253 (0.360)	0.115	NS	0.810	0.624-1.053
		TT	19 (0.054)	35 (0.050)	0.787	NS	0.924	0.520-1.640
		C	525 (0.742)	1083 (0.770)	0.144	NS	1.169	0.948-1.441
		T	183 (0.258)	323 (0.230)			0.856	0.694-1.055
cg03157605	rs9461624	total sample	*357*	*704*				
		GG	325 (0.910)	619 (0.879)	0.162	NS	0.739	0.484-1.130
		TG	32 (0.090)	84 (0.119)	0.182	NS	1.334	0.873-2.040
		TT	0 (0.000)	1 (0.001)	0.476	NS	/	/
		G	682 (0.955)	1322 (0.939)	0.122	NS	0.721	0.476-1.094
		T	32 (0.045)	86 (0.061)			1.386	0.914-2.102
cg16744961	rs10492166	total sample	*358*	*696*				
		GG	123 (0.344)	251 (0.361)	0.584	NS	0.928	0.710-1.212
		GA	169 (0.472)	344 (0.494)	0.495	NS	0.915	0.709-1.181
		AA	66 (0.184)	101 (0.145)	0.098	NS	1.332	0.947-1.871
		G	415 (0.580)	846 (0.608)	0.212	NS	0.890	0.741-1.069
		A	301 (0.420)	546 (0.392)			1.124	0.936-1.350
cg00254095	rs6507921	total sample	*358*	*677*				
		CC	103 (0.288)	232 (0.343)	0.072	NS	1.291	0.977-1.705
		CT	181 (0.506)	308 (0.455)	0.121	NS	0.816	0.632-1.055
		TT	74 (0.207)	137 (0.202)	0.869	NS	0.974	0.709-1.337
		C	387 (0.541)	772 (0.570)	0.196	NS	1.128	0.940-1.353
		T	329 (0.459)	582 (0.430)			0.887	0.739-1.064

**Table 5:** Genotype and allele frequencies of rs10454134 in BD and healthy controls (first and second stage cohorts combined).

Target_ID	CpG-SNP	Genotype	BD n (%)	Controls n (%)	P value	Pc value	OR	95%CI
cg10181180	rs10454134	total sample	*530*	*1034*				
		AA	126 (0.238)	254 (0.246)	0.730	NS	0.958	0.750-1.224
		AG	279 (0.528)	485 (0.469)	0.032	NS	1.258	1.020-1.552
		GG	125 (0.234)	295 (0.285)	0.037	NS	0.773	0.607-0.985
		A	532 (0.502)	993 (0.480)	0.250	NS	1.091	0.941-1.265
		G	528 (0.498)	1075 (0.520)	0.250		0.917	0.791-1.063

## Data Availability

All data generated or analyzed during this study are included in this published article.
